# Abdominal Computed Tomography Imaging Findings in Hospitalized COVID-19 Patients: A Year-Long Experience and Associations Revealed by Explainable Artificial Intelligence

**DOI:** 10.3390/jimaging7120258

**Published:** 2021-12-01

**Authors:** Alice Scarabelli, Massimo Zilocchi, Elena Casiraghi, Pierangelo Fasani, Guido Giovanni Plensich, Andrea Alessandro Esposito, Elvira Stellato, Alessandro Petrini, Justin Reese, Peter Robinson, Giorgio Valentini, Gianpaolo Carrafiello

**Affiliations:** 1 Postgraduation School in Radiodiagnostics, Università degli Studi di Milano, 20122 Milan, Italy; guido.plensich@unimi.it (G.G.P.); elvira.stellato@unimi.it (E.S.); 2 Department of Radiology, IRCCS Fondazione Cà Granda, Ospedale Maggiore Policlinico, 20122 Milan, Italy; massimo.zilocchi@policlinico.mi.it (M.Z.); pierangelo.fasani@policlinico.mi.it (P.F.); gianpaolo.carrafiello@policlinico.mi.it (G.C.); 3 Anacleto Lab, Computer Science Department, Università degli Studi di Milano, 20133 Milan, Italy; elena.casiraghi@unimi.it (E.C.); alessandro.petrini@unimi.it (A.P.); giorgio.valentini@unimi.it (G.V.); 4 CINI National Laboratory of Artificial Intelligence and Intelligent Systems (AIIS), Università di Roma, 00185 Rome, Italy; 5 Department of Radiology, Ospedale Treviglio-Caravaggio, ASST Bergamo-Ovest, 24047 Treviglio, Italy; rxandreaesposito@yahoo.it; 6 Division of Environmental Genomics and Systems Biology, Lawrence Berkeley National Laboratory, Berkley, CA 94720, USA; justinreese@lbl.gov; 7 The Jackson Laboratory for Genomic Medicine, Farmington, CT 06032, USA; Peter.Robinson@jax.org

**Keywords:** COVID-19, SARS-CoV-2, abdominal imaging findings, abdominal symptoms

## Abstract

The aim of this retrospective study is to assess any association between abdominal CT findings and the radiological stage of COVID-19 pneumonia, pulmonary embolism and patient outcomes. We included 158 adult hospitalized COVID-19 patients between 1 March 2020 and 1 March 2021 who underwent 206 abdominal CTs. Two radiologists reviewed all CT images. Pathological findings were classified as acute or not. A subset of patients with inflammatory pathology in ACE2 organs (bowel, biliary tract, pancreas, urinary system) was identified. The radiological stage of COVID pneumonia, pulmonary embolism, overall days of hospitalization, ICU admission and outcome were registered. Univariate statistical analysis coupled with explainable artificial intelligence (AI) techniques were used to discover associations between variables. The most frequent acute findings were bowel abnormalities (*n* = 58), abdominal fluid (*n* = 42), hematomas (*n* = 28) and acute urologic conditions (*n* = 8). According to univariate statistical analysis, pneumonia stage > 2 was significantly associated with increased frequency of hematomas, active bleeding and fluid-filled colon. The presence of at least one hepatobiliary finding was associated with all the COVID-19 stages > 0. Free abdominal fluid, acute pathologies in ACE2 organs and fluid-filled colon were associated with ICU admission; free fluid also presented poor patient outcomes. Hematomas and active bleeding with at least a progressive stage of COVID pneumonia. The explainable AI techniques find no strong relationship between variables.

## 1. Introduction

The coronavirus disease 2019 (COVID-19) pandemic, caused by the severe acute respiratory syndrome coronavirus 2 (SARS-CoV-2), was reported in Wuhan, China, in December 2019 and is still ongoing. It has resulted in more than 104 million cases and over 2.0 million deaths worldwide, as reported by WHO [1].

SARS-CoV-2 is an RNA virus genetically located within the genus Betacoronavirus that uses a glycoprotein (spike protein) to bind to the angiotensin-converting enzyme 2 (ACE2) receptor in order to enter into the cell [2,3]. ACE2 receptors are broadly expressed in the human body viscera, predominantly in type II pneumocytes of pulmonary alveoli. This may result in a wide range of direct targets for the virus, ranging from the lungs to the gastrointestinal system [4].

Accordingly, alongside common clinical features such as fever, cough and myalgia or fatigue, COVID-19 patients may present with and/or develop atypical clinical presentations, including gastrointestinal (GI) symptoms [5].

A multicenter and cross-sectional study demonstrated that approximately 50% of patients experienced symptoms such as diarrhea, nausea, vomiting and abdominal pain [6]. Two recent meta-analyses by Cheung et al. [7] and Parasa et al. [8] showed that the fecal positivity rate for COVID-19 was 40.5% and 48.1%, respectively.

Given the widening recognition of abdominal manifestations, recent studies reported the abdominopelvic findings on CT scans in COVID-19 patients with abdominal symptoms [9,10,11]. Imaging findings may lead to a greater understanding of the pathogenesis of abdominal phenomena in SARS-CoV-2 infection.

The purpose of this study is to assess any association between abdominal CT findings and the radiological stage of COVID-19 pneumonia, the presence of pulmonary embolism and patients’ outcome, in terms of overall days of hospitalization, admission in ICU and patient’s survival/death.

## 2. Materials and Methods

### 2.1. Patients Population

This study was approved by the institutional review board of our Hospital and was in compliance with the Health Insurance Portability and Accountability Act. All aspects of the study were performed in accordance with the Declaration of Helsinki.

We queried our electronic radiologic database to include in our study population all adult patients (>18 year old) who consecutively underwent abdominal CT during their hospitalization between 1 March 2020 and 1 March 2021 and who were positive to real-time reverse transcriptase polymerase chain reaction (RT-PCR) on throat swabs for COVID-19. The demographics and clinical characteristics of the patients were obtained by review of electronic medical records. Age, sex, date of diagnosis, duration of hospitalization, whether the patient was admitted to intensive care unit (ICU) and whether the patient was deceased during the admission were noted.

The evidence of pulmonary embolism on the chest CT executed before or during abdominal imaging was registered.

Stage of COVID-19 pneumonia at the time of abdominal imaging, based only on radiological features in accordance with data from the published literature [12,13], was recorded as follows: no COVID-19 pneumonia (stage 0); early/initial stage (stage 1); progressive stage (stage 2); peak stage (stage 3); absorption stage (stage 4).

### 2.2. Image Acquisition

All CT scans were performed on a 128-slice (Somatom Definition Flash, Siemens AG, Forchheiem, Germany) multidetector CT scanner with or without intravenous contrast media. Scans were obtained during full inspiration in the supine position using the following parameters: 128 × 0.625 mm or 64 × 0.625 mm, tube voltage of 120 kVp and tube current of 180–200 mAs. In patients undergoing contrast material-enhanced CT, axial acquisition of the abdomen and the pelvis was performed after the injection of 80–120 mL of iodinated contrast media (370 mg of iodine per milliliter, Iopamiro, Bracco Imaging Italia s.r.l., Milan, Italy) at different phases (arterial, portal and delayed phases) depending on clinical indication.

### 2.3. Image Analysis

All the available CT scans of the abdomen and pelvis were reviewed by two radiologists in consensus (with 17 and 19 years of experience in radiology) who were blinded to the radiology reports and to clinical data but were aware of positive RT-PCR test for COVID-19.

All pathological findings were noted and classified as acute or not. We considered acute abdominal finding any inflammatory condition, obstruction, thrombosis or hemorrhage that could cause acute symptoms. A subset of patients with inflammatory pathology in organs with high ACE2 receptor expression (bowel, biliary tract, pancreas, urinary system) was identified. Non-acute pathologies such as steatosis, hepatomegaly, cirrhosis, splenomegaly and cancer were also noted.

Dates of examinations and clinical indications were recorded.

All the images were reviewed on Picture Archiving Communication System (PACS, Fuji by Fujifilm Company LTD, Tokyo, Japan).

### 2.4. Explainable AI for Causal Inference

To discover whether the COVID-19 pneumonia stage, pulmonary embolism (PE) and/or admission at intensive care unit (ICU) were associated with any abdominal finding, we applied both univariate statistical tests and explainable artificial intelligence (AI) techniques.

Since the data contained few missing values (the 0.025 of missing values), “AI explanations” were computed over an imputed dataset, where missForest algorithm [14] was used for the imputation. Next, we considered each abdominal finding as a target to be predicted based on linear or complex associations between the following variables: pneumonia stage (stage > 0, stage > 1, stage > 2, stage > 3), PE and/or admission to ICU.

To detect any association between the presence of abdominal findings and the patient outcome (death, number of days of hospitalizations >15, number of days of hospitalizations > 30), we used the abdominal findings as variables to predict any patient outcome.

To produce preliminary explanations and assess individual variables, we initially computed the performance of each variable when used alone in the prediction of any of the outcome variables. To this aim, we used each variable as the unique predictor and assessed it by averaging Sensitivity (Sens), Specificity (Spec), Accuracy (Acc), F1-score (F1), Positive Predictive Value (PPV) and Negative Predictive Value (NPV) over 1000 stratified holdouts with train:test proportion equal to 70:30 (for each integer variable and each train–test split, the best threshold was found on each training set by maximizing the Youden index).

Next, explanations considering variable interactions were produced by averaging the results of 100 repetitions of stratified ten-fold cross-validation, the latest being used in each iteration to obtain unbiased estimates. More precisely, feature selection was initially applied to each training set. To this aim, univariate statistical tests were exploited, where a chi-square test or Wilcoxon rank-signed test at the 95% confidence level (*p*-value < 0.05) were applied for boolean (absence or presence of a specific condition) and integer variables, respectively.

Next, statistically significant variables (*p*-value < 0.05) were used to train eXplainable Artificial Intelligence (XAI) models that can produce explanations in terms of variable importance. More precisely, Logistic Regression classifiers (LR) [15], Generalized Linear Models (GLM) [16], Decision Tree classifiers (DT) [17], Random Forest classifiers (RF) [18] and K-nearest Neighbor classifiers (KNN) [19] were experimented. For GLMs, RFs, DTs and KNNs, the model hyper-parameters were chosen by applying Bayesian optimization [20] operated through an internal 5-fold cross-validation. Each classifier performance was then evaluated by measuring Sensitivity (Sens), Specificity (Spec), Accuracy (Acc), F1-score (F1), Positive Predictive Value (PPV) and Negative Predictive Value (NPV), where classification tasks obtaining an accuracy lower than 0.5 were discarded, since a random choice would, on average, achieve better performance. F1 score, followed by considerations about the balance of sensitivity and specificity, were used to identify the best classifier models. The relative variables’ importance for each prediction task were identified by analyzing the estimated coefficients for LR and GLMs, or the variable importance estimated by using permutation analysis for RFs, DTs and KNNs.

## 3. Results

**Figure 1:**
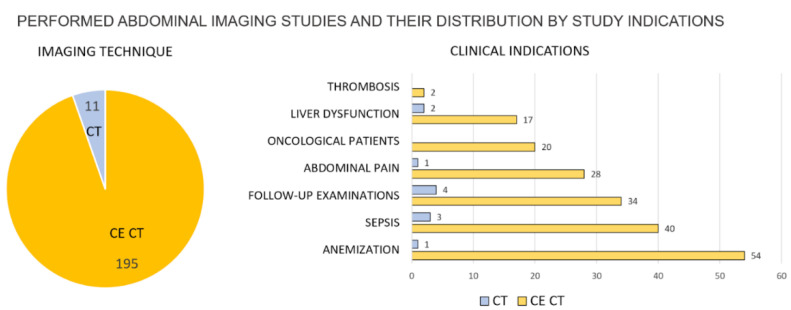
**Left**: Contrast Enhanced Computed Tomography—CE CT (and CTs) are 94.7% (5.3%) of all the acquired CTs. **Right**: study indications for each imaging modality show that anemization is the study indication for which most CE CTs were acquired, while CTs were mostly used for follow-ups. Sixteen patients were found to have pulmonary embolism at chest CT performed before or during abdominal examinations. Embolism was not associated with any abdominal finding (*p* > 0.05).

During the study period, 158 adult patients who tested positive for SARS-CoV-2 underwent 206 abdominal CTs. Patients included 99 men (63%) and 59 women (37 %), with an average age of 66 ± 16 years (range 18 to 95 years). Age and sex were not linearly associated with any abdominal findings. In our cohort of 158 patients, 168 CTs were requested for the onset of abdominal symptoms or changes in the abdominal clinical situation and 38 for follow-up evaluation. The main study indications are summarized in Figure 1.

Radiological stages of COVID-19 pneumonia at time of the 168 abdominal imaging, excluding follow up-studies, were: no COVID-19 pneumonia (stage 0, *n* = 32, 19%) or resolved pneumonia (*n* = 3, 2%), initial pneumonia (stage 1, *n* = 4, 2%), progressive pneumonia (stage 2, *n* = 31, 18 %), peak stages (stage 3, *n* = 45, 27%) and absorption (stage 4, *n* = 53, 32%). According to univariate statistical analysis, for all the different stages (stage > 0, stage > 1, stage > 2) we found a statistically significant difference between the sex distributions (*p*-values ≈ 0), while age distributions was by no means different for different stages (*p*-values > 0.05).

Regarding patient outcomes, 20 (13%) patients were admitted to intensive care units (ICU) and 20 (13%) died. Patients were hospitalized for an average of 38 ± 27 days (range, 3 to 130 days).

The presence of at least one acute pathology in organs with high ACE2 receptor expression was associated with admission to ICU (*p*-value = 0.0238).

We noted 59 bowel abnormalities in 168 abdominal CT images. In 33 patients, the presence of fluid-filled colon suggestive of diarrhea was associated with ICU admission (*p* = 0), days of hospitalization more than 30 (*p* = 0.04) and stage of COVID pneumonia greater than 2 (*p* = 0.02). No associations were found with bowel wall thickening with CT signs of colitis (*n* = 11), enteritis (*n* = 5) or bowel perforation (*n* = 3). A frequent finding was free abdominal fluid (*n* = 42) that was associated with ICU admission (*p*-value = 0) and death (*p* = 0.02).

Other notable abdominal findings were hematomas (*n* = 28) and active bleeding (*n* = 14), which, according to univariate statistical tests, had a significant association with presence of pneumonia stage > 2 (*p* = 0.0051 and *p* = 0.03).

The presence of at least one hepatobiliary finding in 50 patients was associated with the presence of COVID pneumonia (stage > 0).

No associations between abdominal findings and pulmonary embolism were found.

**Figure 2:**
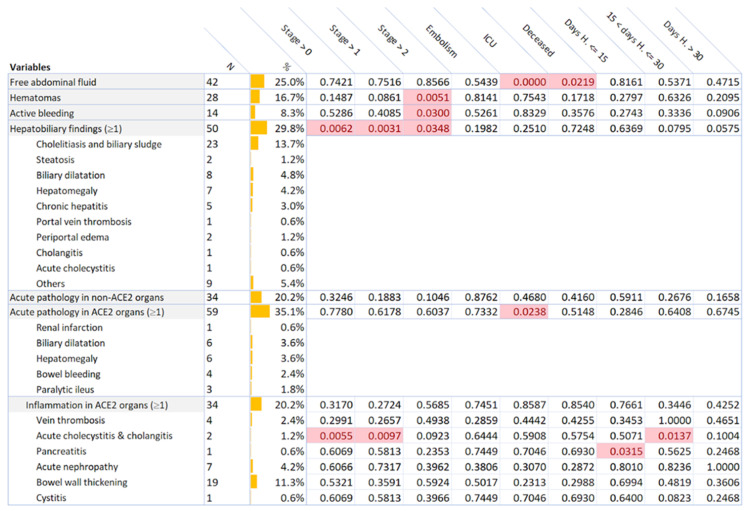
Number of CT abdominal findings, their percentage with respect to all the patients, and the p-value with respect to the patient disease stage, hospitalization history and outcome. Significant values (*p* < 0.05) are highlighted with light red background.

Abdominal imaging findings and related p-values are summarized in Figure 2. In order to improve the reproducibility of the study, their definition is reported in Appendix A.

Furthermore, explainable AI techniques did not find any strong relationships between the analyzed variables (greatest accuracy was 0.7 but with unbalanced sensitivity and specificity).

**Figure 3:**
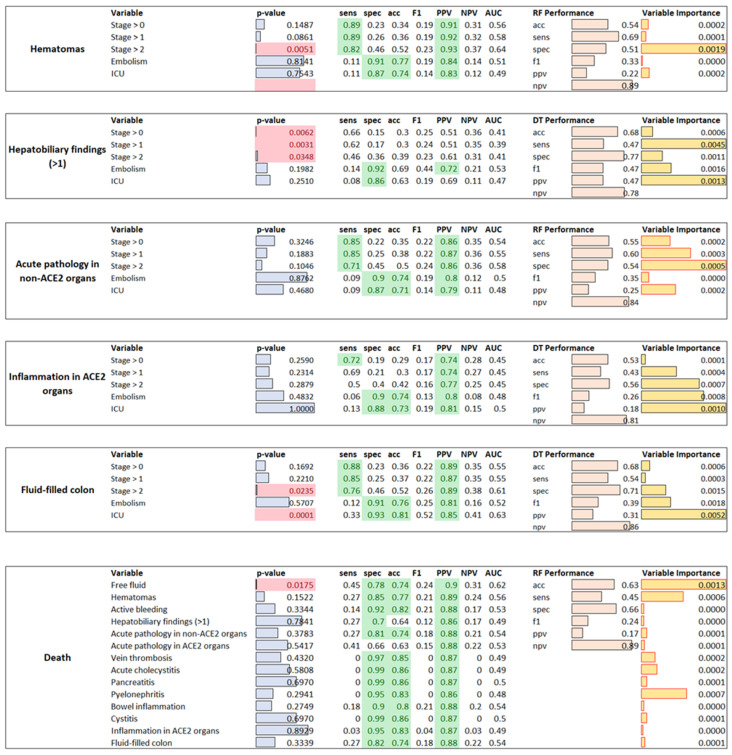
For each variable to be predicted, each sub-table reports the *p*-values obtained by univariate statistical analysis (red color highlights significant variables—*p*-value < 0.05), by the individual predictor variable performance, where green cells highlight good performance (>0.7), the performance of the best classifier model (RF or DT) and the variable importance in prediction.

When applying XAI techniques to understand whether abdominal findings were dependent on the radiological stage of pneumonia or ICU admission, the best predictor of abdominal findings were mostly DT and RF classifiers (see Figure 3).

The only acceptable results (accuracy greater than 0.5 with a balance between sensitivity and specificity) suggesting the existence of weak relationships were obtained for hematomas, dependent only on COVID-19 stage > 2; hepatobiliary findings, dependent on COVD-19 stage > 2; acute pathology in non-ACE2 organs, dependent on all COVID-19 stages in decreased order of severity; inflammations in ACE2 organs, which depended on ICU, embolism and COVID-19 stages in decreasing order of severity; and fluid-filled colon, which depended on admission to ICU and COVID-19 stage > 2.

When predicting patients’ outcome, acceptable classifiers’ performance was obtained only when trying to predict death (RF accuracy = 0.63, sensitivity = 0.45, specificity = 0.66, f1 score = 0.24, ppv = 0.17, npv = 0.89), where free-fluid, hematomas and pyelonephritis were the most important variables. This suggests the existence of weak relationships. Regarding the hospitalization period, univariate tests identified only inflammation in ACE2 organs (*p*-value = 0.0423), acute pathology in non-ACE2 organs (for 15 <= days of hospitalization < 30 *p*-value = 0.0479, days of hospitalization < 30 *p*-value = 0.0236) and hematomas (*p*-value = 0.0209) as significant. Furthermore, considering the low accuracy obtained both by all the classifier models, further tests would be needed to confirm the existence of any, even weak, relationships.

## 4. Discussion

While SARS-CoV-2 has been established as a respiratory tract pathogen, causing non-productive cough, shortness of breath and fever, its pathogenesis may also be responsible for gastrointestinal manifestations, such as diarrhea, nausea, vomiting and abdominal pain [5,6].

Given the widening recognition of abdominal manifestations, recent studies reported the abdominopelvic findings on CT scans in COVID-19 patients with abdominal symptoms [9,10,11].

Following the discovery that the SARS-CoV-2 virus targets host cells expressing ACE-2 receptors [2,3], including the cells of the gastrointestinal tract (certain intestinal cells, cholangiocytes and hepatocytes), major interest has recently been focused on the virus role in dysregulating the digestive system, hepatobiliary function and pancreatic function.

We found that CT signs of acute pathology in abdominal organs with high ACE2 receptor expression were associated with ICU admission.

The presence of at least one hepatobiliary imaging finding was associated with all the progressive COVID-19 pneumonia stages but was not associated with ICU admission, even if these findings are common in critically ill patients admitted in the ICU [21]. However, as recently reported by Nardo et al. [22], hepatobiliary findings may also be related to immune-mediated liver damage and cholangiocellular injury due to the severe inflammatory response/systemic inflammatory response syndrome (SIRS) in COVID-19. Chai et al. [23] found that ACE2 expression levels in bile duct cells were slightly higher than those in liver cells and were comparable with alveolar epithelial type II cells; these expression levels revealed the possibility for direct infection of cholangiocytes by SARS-CoV-2. They also suggested that liver abnormalities of COVID-19 patients may be due cholangiocyte dysfunction since cholangiocytes play critical roles in liver regeneration and immune responses [24]

In Shiralkar et al.’s study [10], bowel abnormalities, including acute pancreatitis and acute cholecystitis, were seen in 25% of patients. In our study, 27% of patients had bowel abnormalities, but there was only one case of pancreatitis, acute cholecystitis and cholangitis.

Similarly, Bhayana et al. demonstrated a spectrum of abdominopelvic imaging findings that correlated with COVID-19 infection with the predominant imaging findings of bowel wall thickening (31%) [11]. The incidence of bowel abnormalities was also high in the study by Tirumani et al., where abnormal bowel wall was seen in 18.1% of cases, fluid-filled colon in 16.7% of patients and severe colitis was seen only in one patient (1.4%) [9]. We found that a fluid-filled colon was significantly associated with ICU admission, a longer period of hospitalization (more than 30 days) and a progressive stage of COVID pneumonia (>2). Moreover, the presence of free abdominal fluid, which was a frequent finding, was associated with ICU admission and death.

Two recent meta-analyses showed that there is a high incidence of fecal positivity rate in COVID-19 infection (nearly 40–48%), though the prevalence of GI symptoms is seen only in a few patients (4–12%) [7,8].

A recent study by El Moheb et al. showed that a higher rate of gastrointestinal complications was found in critically ill COVID-19 patients compared with propensity score-matched patients without COVID-19 [25].

ACE2 receptors are expressed in enterocytes of the small intestine and vascular endothelium, suggesting that the small bowel and vasculature may be susceptible to SARS-CoV-2 infection [2,3]. However, there are still many open questions about the potential pathophysiological mechanisms for SARS-CoV-2 bowel tropism.

In our study, no associations were identified between bowel wall thickening and the presence of COVID pneumonia, pulmonary embolism or patient’s outcome. However, in four patients, endoscopic biopsy identified ischemic mucosal necrosis, indicating that SARS-CoV-2 might have played an active role in relevant intestinal hypoperfusion, but no vascular causes were clearly identified at CT. Funt et al. [26] also depicted microbial metabolites and endotoxins released into the blood from lung inflammation can alter the microbiome of the gut leading to abdominal symptoms in the so-called “gut-lung” axis.

In our study, according to univariate statistical analysis, COVID-19 pneumonia stage > 2 defined at the time of abdominal imaging acquisition was significantly associated with increased frequency of at least one hepatobiliary finding, hematomas, active bleeding and a fluid-filled colon.

ACE2 receptors are expressed on kidney podocytes and proximal convoluted tubules cells, suggesting a potential role of SARS-CoV-2 infection in the pathophysiology of acute renal failure due to a direct virus-induced cytopathic effect [27]; however, in our cohort of patients, no statistically significant association was found.

Zaim et al. [28] reported that up to 19% of patients with COVID-19 have acute renal dysfunction. This must be taken into consideration before administering contrast agents for CT and MRI studies in COVID-19 patients.

In our study, no statistically significant associations were found between COVID-19 patients who developed pulmonary embolism (PE) during their hospitalization and acute inflammatory findings in abdominal organs with high ACE2 receptors. Currently, in coronavirus disease (COVID-19), the origin of endothelial dysfunction that may lead to PE is not known. As reported by Rodriguez et al. [29], the endothelium of pulmonary arteries appears to be highly sensitive to SARS-CoV-2 infection, given its expression of the ACE-2 receptor. However, endothelial dysfunction could also occur secondarily to the activation of inflammatory and/or coagulation and complement cascades [29]. We theorize that this second mechanism may explain better the absence of association with acute inflammatory imaging findings in abdominal organs with high ACE2 receptors. The limitations of the study include its retrospective nature, the small sample size, the absence of pathologic correlation and the lack of fecal COVID-19 testing.

There are still many questions about the mechanisms underlying the pathophysiology of SARS-CoV-2 infection, and larger prospective studies with histopathologic correlation are required to understand the pathogenesis of these abdominopelvic imaging findings better [9,10,11].

## 5. Conclusions

### Abdominal Abnormalities Were Common Findings in COVID-19 Patients

We found some statistical associations between abdominal findings and patients’ outcomes or stages of COVID pneumonia. In particular, the presence of acute pathology in abdominal organs with high ACE2 receptors was significantly associated with ICU admission. Moreover, explainable AI techniques did not find any strong relationships between the analyzed variables; therefore, further investigations are required.

## Data Availability

The data presented in this study are available on request from the corresponding author.

## Appendix A

To improve the reproducibility of our study, in this appendix, we provide the main definitions (and their source) for the abdominal image findings we identified in the patient cohort used in this study.

Acute cholecystitis [30]: Acute cholecystitis is one of the complications of cholelithiasis due to stones getting impacted and persistently obstructing the cystic duct. This results in inflammation and in the consequent release of inflammatory enzymes, which worsen damages in the mucosa and eventually cause ischemia, promote bacterial infection and may ultimately result in necrosis and perforation. Acute cholecystitis causes a fibrotic and contracted gallbladder, unable to concentrate bile and empty it.

Acute nephropathy or Acute renal failure [31,32]: Acute renal failure is due to a rapid fall in the rate of glomerular filtration, which manifests clinically as an abrupt and sustained increase in the serum levels of urea (by >0.3 mg/dL (X26.5 lmol/L) within 48 h) and creatinine (to >1.5 times baseline) with an associated disruption of salt and water homeostasis and a reduced urine volume (<0.5 mL/kg/h for 6 h).

Biliary dilatation (dilation) [33]: Bile is a substance (produced by the liver and accumulated in the gallbladder) that is involved in the digestion of fats. For this reason, it is brought into the intestines through the bile ducts (biliary ducts) when food is ingested. As a consequence of injury or surgery, the biliary ducts may become narrower and narrower until becoming stuck. In this case, the procedure of biliary dilation has the effect of reopening biliary ducts to avoid consequences such as bile duct inflammation (cholangitis), liver abscess or secondary cirrhosis.

Bowel bleeding [34]: Gastrointestinal bleed includes upper GI tract bleeds (about 50% of GI bleeds), lower GI tract bleeds (40%) and bleeds due to vascular lesions in the small bowel (about 5% to 10%). Clinically, it manifests with visible bleeding, either melena or hematochezia, or occult bleeding, when there is no gross bleeding, but signs and symptoms of anemia (fatigue, dyspnea or palpitations) are present. The management options for GI bleeding involve conservative, radiological, pharmacologic, endoscopic and surgical methods.

Bowel wall thickening [35]: The normal bowel wall measures between 1 and 2 mm for the small bowel and less than 3 mm for the colon. However, it must be considered that the thickness of the normal bowel wall can vary depending on the degree of luminal distention. Bowel wall thickening may be due to normal variants, inflammatory conditions and neoplastic disease.

Cholangitis [36]: Acute cholangitis is caused by an ascending bacterial infection of the biliary tree, usually due to the presence of stones in the common bile duct, that leads to partial or complete obstruction of the biliary system, with an increased risk of superinfection. If not treated, it can lead to septic shock. Depending on the course and severity, a biliary drainage procedure may be performed.

Cholelithiasis and biliary sludge [37]: Cholelithiasis is the presence of one or more stones in the gallbladder. It is usually asymptomatic; when symptomatic, it manifests with biliary colic. More serious complications include biliary tract obstruction, infection (cholangitis and/or cholecystitis) and gallstone pancreatitis.

Biliary sludge is made of calcium bilirubinate (a polymer of bilirubin), cholesterol microcrystals and mucin. It develops during gallbladder stasis, and it is often a precursor of gallstones.

Chronic hepatitis [38]: Chronic hepatitis is defined as hepatitis that lasts over 6 months. It can be caused by hepatitis B and C viruses, nonalcoholic steatohepatitis (NASH), alcohol-related liver disease and autoimmune liver disease (autoimmune hepatitis). It may present with cirrhosis or its complications, such as portal hypertension. Treatment varies upon the underlying condition. Liver transplantation can be indicated.

Cystitis [39]: Cystitis is the inflammation of the lining of the bladder. Common causes include urinary infection, medicines (such as anticancer drugs) or chemicals (such as perfumes or dyes), radiation therapy, long-term urinary catheterization and underlying conditions, such as diabetes, kidney stones, enlarged prostate or a spinal cord injury. Clinically, it manifests with pain and a burning feeling while urinating, blood in the urine and dark or cloudy urine.

Hematoma [40]: Hematoma is defined as a pool of mostly clotted blood in an organ, tissue or body space. It is caused by a vascular lesion due to surgery or an injury that leads to blood leaking into the surrounding space. Hematomas may resolve spontaneously over time as the blood debris is removed. Surgically removing or evacuating the blood becomes necessary based on its symptoms or location.

Hepatomegaly [41]: Hepatomegaly is an enlarged liver. Its size varies with sex and body size, but it usually extends from the fifth intercostal space to the right costal margin in the midclavicular line. By ultrasound, a liver span >16 cm in the midclavicular line is indicative of hepatomegaly.

Pancreatitis [42]: Acute pancreatitis is an acute response to injury of the pancreas. The most common causes include gallstones (35% to 40% of cases), alcohol use (30% of cases), autoimmune pancreatitis, hypertriglyceridemia, post-endoscopic retrograde cholangiopancreatography (ERCP), genetic risk, pancreatic duct injury, medication and drugs.

Chronic pancreatitis is defined as a continuing inflammatory disease of the pancreas, characterized by irreversible morphologic changes that can result in permanent organ damage. Alcohol has been identified as a definitive cause of chronic pancreatitis.

Paralytic ileus [43]: Paralytic ileus is characterized by the dilation of the bowel in the absence of mechanical obstruction. Clinically, it manifests with nausea, vomiting, abdominal distension and obstipation; on x-ray or CT imaging with bowel dilation. Causes are usually infectious, metabolic, neurologic (linked to dysregulation of the autonomic nervous system), autoimmune, iatrogenic (recent surgery) or idiopathic.

Periportal edema [44]: Periportal edema is related to passive congestion or diminished perfusion of the liver, usually attributable to hepatic injury. It can be associated with Budd-Chiari syndrome, hepatic sinusoidal obstruction syndrome, passive congestion due to heart failure, hepatic infarction and ischemic hepatitis.

Portal vein thrombosis [45]: Portal vein thrombosis is the occlusion of the portal vein (formed by the confluence of the splenic and the mesenteric veins, which drain the spleen and the intestine, respectively) by a thrombus (clotted blood). It typically occurs in patients with cirrhosis and/or prothrombotic disorders. When chronic, it can lead to collateral circulation (e.g., cavernous portal transformation) or portal hypertension.

Renal infarction [46]: Renal infarction results from interruption of the arterial supply (either the main renal artery or the smaller segmental branch arteries) to the kidney. Common causes include cardioembolic disease, renal artery injury (e.g., arterial dissection), hypercoagulable states, thromboembolism and in situ thrombosis. Depending upon the severity, renal infarction can lead to renovascular hypertension, chronic kidney disease and end-stage kidney disease.

Steatosis [47]: Steatosis is defined as fatty infiltration of the liver due to insulin resistance associated with central obesity and a sedentary lifestyle. In the early stages, it is reversible. If not treated, steatosis may progress to steatohepatitis, which can lead to progressive hepatic injury and fibrosis.

Vein thrombosis [48]: A vein thrombosis is a blood clot (thrombus) that forms within the veins. It is usually associated with increased blood viscosity, reduced blood flow, increased venous pressure, genetic deficiencies and acquired and constitutional factors.
